# Chloroquine/Sulphadoxine-Pyrimethamine for Gambian Children with Malaria: Transmission to Mosquitoes of Multidrug-Resistant Plasmodium falciparum

**DOI:** 10.1371/journal.pctr.0010015

**Published:** 2006-07-21

**Authors:** Rachel L Hallett, Samuel Dunyo, Rosalynn Ord, Musa Jawara, Margaret Pinder, Anna Randall, Ali Alloueche, Gijs Walraven, Geoffrey A. T Targett, Neal Alexander, Colin J Sutherland

**Affiliations:** 1 Immunology Unit and Infectious Diseases Epidemiology Unit, Department of Infectious and Tropical Diseases, London School of Hygiene and Tropical Medicine, London, United Kingdom; 2 Farafenni Field Station, Medical Research Council Laboratories, Fajara, The Gambia

## Abstract

**Objectives:**

In the Gambia, chloroquine (CQ) plus sulphadoxine-pyrimethamine (SP) is the first-line antimalarial treatment. Plasmodium falciparum parasites carrying mutations associated with resistance to each of these drugs were present in 2001 but did not cause a significant loss of therapeutic efficacy among children receiving the combination CQ/SP. We measured their effect on parasite transmission to Anopheles gambiae mosquitoes.

**Design:**

We conducted a single-blind, randomised, controlled trial with follow-up over 28 d. Mosquito feeding experiments were carried out 7, 10, or 14 d after treatment.

**Setting:**

The study took place in the town of Farafenni and surrounding villages in the Gambia.

**Participants:**

Participants were 500 children aged 6 mo to 10 y with uncomplicated P. falciparum malaria**.**

**Interventions:**

Children were randomised to receive CQ, SP, or CQ/SP.

**Outcome Measures:**

Outcomes related to transmission were determined, including posttreatment gametocyte prevalence and density. Infectiousness was assessed by membrane-feeding A. gambiae mosquitoes with blood from 70 gametocyte-positive patients. Mutations at seven loci in four genes associated with drug resistance were measured pre- and posttreatment and in the midguts of infected mosquitoes.

**Results:**

After SP treatment, the infectiousness of gametocytes was delayed, compared to the other two treatment groups, despite comparable gametocyte densities. Among bloodmeal gametocytes and the midguts of infected mosquitoes, the presence of the four-locus multidrug-resistant haplotype TYRG (consisting of mutations *pfcrt*-76T, *pfmdr1*-86Y, *pfdhfr*-59R, and *pfdhps*-437G) was associated with significantly higher oocyst burdens after treatment with the combination CQ/SP.

**Conclusions:**

Parasites with a multidrug-resistant genotype had a substantial transmission advantage after CQ/SP treatment but did not have a significant impact on in vivo efficacy of this drug combination. Protocols that include measuring transmission endpoints as well as therapeutic outcomes may be a useful strategy when monitoring the evolution of drug resistance in malaria parasites in vivo.

## Editorial Commentary

**Background:** In an accompanying paper published in *PLoS Clinical Trials* [15], the investigators report the effects of treating uncomplicated malaria in young children in the Gambia with the combination of chloroquine and sulfadoxine-pyrimethamine, as compared to either treatment individually. As an addition to the main trial results, the researchers also wanted to find out how the different treatment regimens affected the ability of the malaria parasite to generate sexual forms (gametocytes) in the participants' blood, and to what extent the parasites are then transmitted to mosquitoes. The researchers also tested whether malaria parasites had a specific genotype, termed TYRG, comprising mutations in four distinct genes associated with resistance to chloroquine and sulfadoxine-pyrimethamine, and whether that affected transmission of the parasites back to mosquitoes.

**What the trial shows:** In this trial it was found that children treated with chloroquine and sulfadoxine-pyrimethamine were no more or less likely to carry sexual forms of the malaria parasite compared to children treated with chloroquine, over 28 days of follow-up. Transmission of the malaria parasites back to mosquitoes also did not differ significantly between children treated with chloroquine and sulfadoxine-pyrimethamine versus chloroquine alone. Parasites bearing the TYRG genotype, associated with drug resistance, were more likely to be transmitted to mosquitoes from children treated with chloroquine/sulfadoxine-pyrimethamine than from chloroquine treated children.

**Strengths and limitations:** The addition of data about parasite transmission within the setting of a randomized trial is valuable and can provide information about the evolution of drug resistance. However, the results may not completely represent the current situation, as the trial was originally conducted in 2001 and levels of resistance may now be higher in the Gambia.

**Contribution to the evidence:** This follow-up study to a randomized trial provides additional evidence that treatment with chloroquine and sulfadoxine-pyrimethamine, as compared to chloroquine, does not reduce the extent to which malaria parasites can produce sexual forms and transmit them back to mosquitoes. The finding that parasites bearing the TYRG genotype are more likely than other parasites to be transmitted back to mosquitoes following chloroquine and sulfadoxine-pyrimethamine treatment is also novel.

## INTRODUCTION

The widespread occurrence of drug-resistant parasites in malaria endemic regions has led to rapid changes in antimalarial treatment policies in many countries. In some parts of Southeast Asia, multidrug-resistant parasites are prevalent and have greatly reduced the efficacy of the majority of monotherapy regimens. This has led to the implementation of combination therapies, including artemisinin-based treatment throughout the region [1]. In sub-Saharan Africa, monotherapies such as chloroquine (CQ) and the antifolate fixed combination sulphadoxine-pyrimethamine (SP) have continued to be widely used. Parasites resistant to CQ are common and known to contribute to excess severe malaria cases [2], and SP-resistant genotypes have spread widely across the continent from a few focal origins [3]. Thus, despite there being few studies of multidrug-resistant Plasmodium falciparum in Africa, it is likely that parasites harbouring mutations conferring resistance to both CQ and SP circulate in many areas. Policy changes towards the implementation of artemisinin-containing combination therapies (ACTs) are now occurring in many countries [4].

One of the potential benefits of ACT use is that the artemisinin component has the demonstrated ability to reduce gametocyte carriage and thus transmission to mosquitoes [5]. Other antimalarial drugs vary in their effect on gametocytes; CQ and other 4-aminoquinolines exert their effect on the parasite's haem-metabolising stages, which includes early-stage gametocytes. In contrast, antifolates such as SP are only effective during asexual development and will not affect parasites once these are committed to the sexual pathway [6].

The implementation of ACTs is costly and is currently beset with problems of supply due to the very rapid expansion in demand for artemisinin drugs in the last few years. Therefore, some African Ministries of Health have moved to combinations of established antimalarials as a first step away from monotherapies, in anticipation of a second switch to ACTs as soon as it is feasible. Senegal has implemented SP plus amodiaquine as first-line replacement for CQ monotherapy, with a move to amodiaquine plus artesunate planned for 2006. In the Gambia, where CQ monotherapy is no longer efficacious [7], it has been replaced by CQ combined with SP (CQ/SP), with the longer-term goal being implementation of ACT.

Earlier studies in the Gambia had shown that CQ plus SP was as efficacious as SP alone as treatment for uncomplicated malaria in children and was more effective at providing rapid symptomatic relief [8]. However, in studies of the infectiousness to mosquitoes of treated Gambian children with uncomplicated falciparum malaria, we have found that SP and CQ both permit substantial transmission during the first week following treatment [9,10] as does the combination CQ/SP [11]. CQ-resistant parasites were already highly prevalent in the Farafenni area when these studies were carried out and displayed enhanced gametocyte carriage and higher intensity of mosquito infection than wild-type parasites in CQ-treated individuals [12,13]. Furthermore, we have shown that mutant forms of the *pfdhfr* and *pfdhps* loci associated with SP resistance and carriage of gametocytes after SP treatment [14] were also common [15]. We were concerned therefore that the combination CQ/SP would selectively enhance the transmission and circulation of multidrug-resistant parasites. Such an enhancement may persist for some time after treatment, given the long half-life of SP, and the longevity of P. falciparum gametocytes.

In 2001, we conducted a single-blind, randomised, controlled trial of CQ versus SP versus CQ/SP in Gambian children aged 6 mo to 10 y presenting to Farafenni Hospital with uncomplicated P. falciparum malaria. We report in the accompanying paper [15] the therapeutic efficacy of the regimens used, and the identification of a four-locus parasite genotype that was associated with treatment failure and may represent a multidrug-resistant phenotype in the Gambia. Posttreatment gametocyte carriage and infectiousness of gametocyte carriers to mosquitoes are examined here. We also evaluated the contribution of parasites carrying a multidrug-resistant genotype to transmission success in each treatment group.

## METHODS

The study was a single-blind, randomised, controlled trial to compare efficacy of treatment of children with uncomplicated P. falciparum malaria with CQ, SP, or CQ/SP. Full details of the participants and interventions are described in the accompanying paper [15].

### Objectives

The primary objectives were to measure posttreatment gametocyte prevalence and density in each of the three treatment groups and to assess how this related to infectiousness to mosquitoes. Within the SP group, the effect of time after treatment was also examined, because gametocyte samples were collected on three different follow-up days. Secondary measurements included the contribution of genetically defined drug-resistant parasites to mosquito oocyst burden and the effect of drug group on transmission success.

### Outcomes

Outcomes measured were gametocyte carriage and density on defined follow-up days, and the proportion of mosquitoes infected. The number of oocysts was counted in each infected mosquito midgut. For all infections used for mosquito feeding, the genotype at drug resistance-associated loci was determined at three time points: pretreatment, and posttreatment in the gametocyte-positive blood feed sample and in the mosquito midguts arising from successful infections.

### Sample Size and Randomisation

The sample size, randomisation, and blinding are described in the accompanying paper [15].

### Posttreatment Follow-Up

Follow-up procedures relating to efficacy endpoints are described in the accompanying paper [15]. In order to fulfill the transmission objectives, children were collected from their homes either on day 7, 10, or 14 after treatment and taken to the Medical Research Council Field Station in Farafenni, where they were clinically examined and finger prick blood samples were obtained for thick blood film preparation and packed cell volume estimation. The blood films were stained with Field's stain and examined immediately for malaria parasites. Children with gametocytes were requested to provide 3.0 ml of venous blood for mosquito infectivity experiments as previously described [9,10]. For those who had received CQ or CQ/SP, this happened on day 7 only. Among the SP-treated group, children were assigned a gametocyte screening day according to the day of the week they were recruited to overcome the logistical problems that would be caused by a nested second randomisation. Thursday and Friday recruits receiving SP were screened for gametocytes on day 7, Tuesday and Saturday recruits were screened on day 10, and Monday and Wednesday recruits were screened on day 14. Recruiting did not occur on Sundays. SP-treated children who were screened on day 10 or 14 were not visited on day 7 and therefore were not subject to follow-up equivalent to that of SP-treated children screened on day 7 or of children in the other treatment groups, all of whom were screened on day 7.

### Membrane-Feeding and Mosquito Dissection

Venous blood samples were obtained from children with a packed cell volume of >20%, who had been free of peripheral gametocytes at the time of recruitment (day 0) but who were gametocytaemic (limit of detection, 5 gametocytes/μl) when screened on day 7, 10, or 14. Specific consent for the procedure was obtained from the parents or guardians. If no children fulfilling these criteria were available as donors but cages of mosquitoes had been prepared, children who had been gametocyte positive on day 0 were selected. Membrane-feeding was performed as described elsewhere [9,10]. In brief, venous blood in citrate-phosphate dextrose was centrifuged, and the plasma was removed. After being washed, the red blood cell pellet was split into two aliquots of 300–500 μl each. These were resuspended to a packed cell volume of 33% in, respectively, the original autologous plasma and in pooled AB serum from European donors with no history of malaria exposure (control serum). Each suspension was then fed to approximately 50 3- to 5-d-old female Anopheles gambiae mosquitoes (obtained as F_1_ progeny of wild-caught gravid females) via an artificial membrane attached to a water-jacketed glass feeder maintained at 37 °C. Mosquito midguts were dissected out 7–8 d later, and the number of malaria oocysts on each one was recorded. Each oocyst-positive midgut was retained in a separate tube of 95% ethanol.

### Parasite Genotyping

DNA was extracted from day 0 bloodspots on filter paper and from the remainder of the venous blood sample taken for membrane-feeding by boiling with chelex-100 [16]. Oocyst DNA was obtained from infected mosquito midguts as follows: each sample was rehydrated in 500 μl of a 1:1 mix of ethanol and TE buffer (10 mM Tris-HCl, 1 mM EDTA) before being incubated at 56 °C overnight in 200 μl of 6% chelex containing 20 μg of proteinase K. Samples were then boiled for 30 min and 5 μl of the supernatant was used in PCR amplifications [17].

A sequence-specific oligonucleotide probing assay was used to detect polymorphisms at loci associated with CQ and SP resistance in all PCR-positive DNA extracts [12,18]. These were *pfcrt*-K76T, *pfmdr1*-N86Y, *pfmdr1*-Y184F (linked to CQ resistance), and *pfdhfr*-N51I, *pfdhfr*-C59R, *pfdhfr*-S108N, *pfdhps*-A437G, and *pfdhps*-K540E (linked to SP resistance). For some analyses, where mixtures of drug-sensitive and drug-resistant parasites were detected, they were scored as resistant to reflect the expected phenotype of the infection.

### Statistical Methods

All data were double entered and verified using Epi-Info, version 6 (Centers for Disease Control and Prevention, Atlanta, Georgia, United States). Clinical, parasitological, and entomological data entered in Epi-Info were transferred to Stata 8.1 (Stata Corporation, College Station, Texas, United States) for statistical analysis. Categorical variables were compared among groups using odds ratios and significance tested by the Fisher exact test. Gametocyte densities counted in thick films and oocyst density on mosquito midguts were compared between treatment groups in pairwise fashion. The ratio of the arithmetic means was calculated, and generalized linear models with a logarithmic link function and negative binomial distribution family were fitted to the data. This models the logarithm of the (arithmetic) mean gametocyte density, or oocyst density as previously described [10,11]. Ratios of mean densities were obtained by exponentiating the relevant regression coefficients. Oocyst data were corrected for clustering within mosquito cages fed on blood from the same individual.

## RESULTS

### Participant Flow

**Figure 1:**
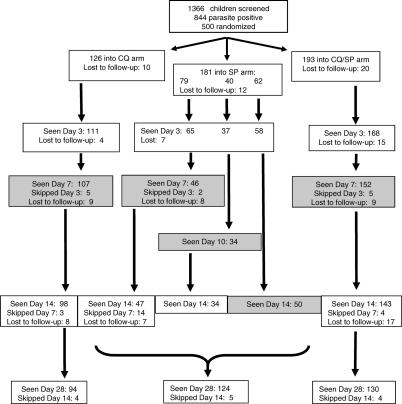
CONSORT Flowchart A total of 1,366 children was screened, and 500 were randomised. Children randomised to receive SP were scheduled for gametocyte screening on day 7, 10, or 14 (see text). Shaded boxes represent gametocyte screening days. Loss to follow-up is detailed in the main trial paper [15].

A total of 500 eligible patients were enrolled into the trial, as described in detail elsewhere [15]. After allocation to treatment groups, 193, 181, and 126 children were treated with CQ/SP, SP, and CQ, respectively. Among the SP-treated group, 79, 40, and 62 children were scheduled for gametocyte screening on days 7, 10, and 14, respectively. A trial profile is presented in Figure 1, and clinical and parasitological outcomes are presented in the accompanying paper [15].

### Gametocyte Prevalence and Density

**Figure 2:**
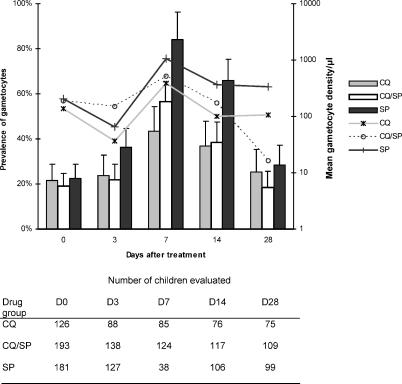
Prevalence and Positive Arithmetic Mean Density of Gametocytes in Treated Children Those carrying gametocytes at day 0 were excluded from the denominator at subsequent follow-up days (see data table). Bars represent prevalence of gametocyte carriage, with error bars showing 95% CIs. Lines represent arithmetic mean gametocyte density in carriers only.

The prevalence of posttreatment gametocyte carriage at each day of follow-up among children without gametocytes at presentation is shown in Figure 2. Approximately 20% of children in each treatment group were gametocyte-positive at day 0. This is consistent with our previous findings [10]. These children, who had a 90% chance of carrying gametocytes at some point during follow-up after treatment (data not shown), are omitted from prevalence estimates at subsequent time points. In pairwise comparisons, there was no significant difference in gametocyte carriage between the CQ and CQ/SP treatment groups at day 3 (*p* = 0.71), day 7 (*p* = 0.066), day 14 (*p* = 0.82), or day 28 (*p* = 0.26). In contrast, SP-treated children were significantly more likely to become gametocyte carriers than were CQ/SP-treated or CQ-treated children at day 3 (*p* = 0.009, 0.054, respectively), day 7 (*p* = 0.002, < 0.001), and day 14 (*p* < 0.001 in both cases), but not at day 28 (*p* = 0.089, 0.66).

Gametocyte carriage in the CQ treatment group was closely associated with treatment failure, as previously observed in this population [10,12]. Gametocyte carriage during follow-up was observed in 14 of 39 children (35.9%) presenting free of gametocytes and successfully treated with CQ, compared to 40 of 53 children (75.5%) who carried P. falciparum trophozoites at any point during the 28 d of follow-up (odds ratio, 5.17; 95% confidence interval [CI], 1.95–13.9; *p* < 0.0001). This was not seen in either of the other treatment groups, which were both highly efficacious [15].

A pairwise comparison of mean gametocyte density between treatment groups at each time point was carried out, including all evaluable children who were not gametocyte carriers at presentation. Gametocyte density in the SP-treated group was significantly higher than in the CQ/SP group at days 7, 14, and 28 (ratio of means, 3.06 [*p* = 0.031], 3.68 [*p* = 0.001], and 31.2 [*p* < 0.001], respectively). Gametocyte density in the CQ/SP-treated group was higher than in the CQ group at day 3 (ratio of means, 3.76 [*p* = 0.046]), but there was no significant difference between these two groups at day 7 (ratio of means, 1.73 [*p* = 0.30]) or day 14 (ratio of means, 1.69 [*p* = 0.25]). At day 28, gametocyte density in the CQ/SP-treated group was significantly lower than in the CQ group (ratio of means, 0.111 [*p* = 0.001]).

### Infectiousness of Gametocyte Carriers

Venous blood was donated by 72 gametocyte carriers for membrane-feeding, resulting in 70 evaluable feeding experiments, and the dissection of 2033 blood-fed adult female mosquitoes. Results of the feeding experiments in both autologous plasma and control serum are presented in Table 1.

**Table 1:**
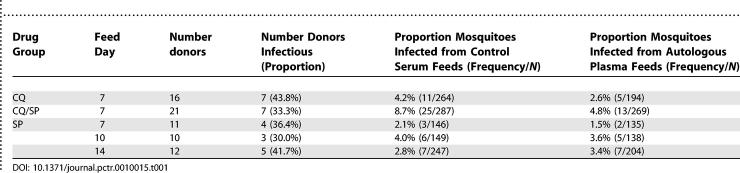
Results of Membrane-Feeding Experiments

**Figure 3:**
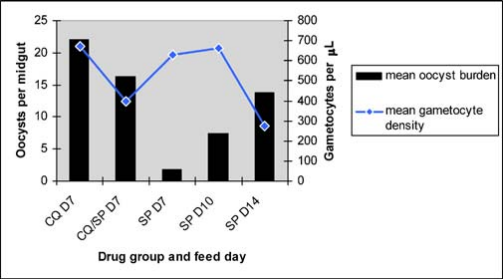
Relationship between Gametocyte Density and Oocyst Burden in Feed Samples

As a measure of infectiousness, the mean number of oocysts per infected midgut was compared among treatment groups and across different feed days in the SP-treated group (Figure 3 and Table 2). In regression analysis, the mean oocyst burden did not differ significantly between the CQ and CQ/SP groups (data not shown). Among SP-treated donors, those identified 10 or 14 d after treatment were significantly more infectious than were day 7 donors (*p* = 0.008, *p* = 0.020 respectively), despite high gametocyte densities in the latter group (Figure 3 and Table 2).

**Table 2:**
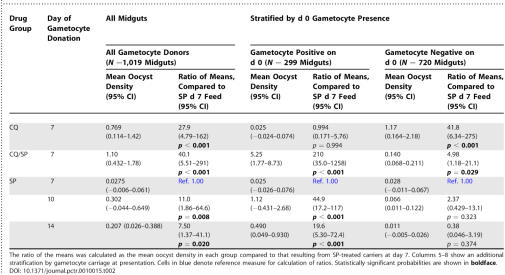
Mean Oocyst Densities after Feeds from All Gametocyte Carriers at d 7 after Treatment, and in SP-Treated Carriers at Days 10 and 14

When we compared SP-treated children who carried gametocytes on day 0 with those who did not, significantly greater infectiousness in day 10 and 14 gametocyte carriers was seen only among those with gametocytes at presentation (Table 2). There is therefore an important contribution of gametocyte maturity to infectivity.

### Contribution of Resistant Genotypes to Circulating Gametocyte Populations

We report elsewhere on the prevalence of resistance-associated parasite genotypes in the study population, and the contribution of such parasites to treatment failure [15]. It was found that multidrug-resistant parasites exemplified by the four-allele genotype TYRG (i.e., simultaneous carriage of *pfcrt*-76T, *pfmdr1*-86Y, *pfdhfr*-59R, *pfdhps*-437G) were contributing significantly to parasitological failure after treatment with SP. Parasites with the TYRG genotype were also present in some cases after treatment with CQ/SP and thus likely to be selected with widespread combination drug pressure. We therefore tested for associations between either posttreatment gametocyte emergence or transmission success and resistance-associated alleles at seven loci alone and in combination.

**Figure 4:**
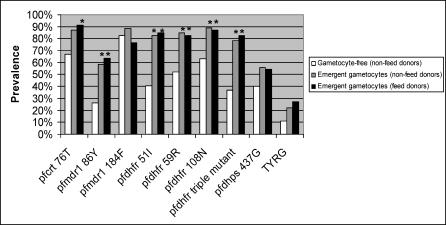
Contribution of Resistance-Associated Parasite Genotypes at Day 0 to Subsequent Gametocyte Emergence Only subjects free of circulating gametocytes at enrollment are included. Pretreatment genotype data were obtained for 27 patients who remained gametocyte-free during follow-up (white bars) and 46 patients with emergent gametocytes (grey bars). Pretreatment genotype prevalences for 47 gametocyte feed donors are shown by the black bars. * indicates a significantly higher prevalence of the designated genotype than among the baseline nongametocyte carriage at presentation.

Carriage of genetically resistant parasites at presentation was associated with the presence of gametocytes during the follow-up period, as shown in Figure 4. Gametocyte donors carried higher pretreatment prevalences of CQ and SP resistance-associated markers than a random sample of the trial population who had remained gametocyte negative. This increase was significant for the CQ resistance markers *pfcrt*-76T and *pfmdr1*-86Y, but not for *pfmdr1*-184F. Similarly, the difference was significant when analysing the pyrimethamine-resistant mutations in the *pfdhfr* gene (codons 51, 59, and 108) both individually and together as presence of the “triple mutant.” *Pfdhps*-437G was the only sulphadoxine-resistant mutation detected in this population, and was found at a higher prevalence in gametocyte donors at day 0, but this difference was not significant. When grouping mutations into the multidrug resistance-associated genotype TYRG as defined in Dunyo et al. [15] we found that this combination of mutations showed an association with posttreatment gametocytaemia (odds ratio, 3.0; 95% CI, 0.78**–**17.0; two-sided Fisher exact, *p* = 0.11), although this was also not statistically significant.

### Selection of Drug Resistance Markers among Gametocyte Donors

We have previously presented evidence of within-host directional selection from *pfcrt*-76K to T after CQ treatment [13]. However, in the current dataset, too few genotype changes occurred between recruitment and mosquito-feeding to perform this analysis for any of the loci examined. Resistance markers were highly prevalent in presentation samples and, in general, genotypes detected in gametocyte-positive feed samples and in resulting oocysts reflected what was present at day 0 (Table S1).

In two feeds (MF27 and MF40), mixed infections were detected in some midguts, but not others, at the *pfdhfr* and *pfdhps*-437 sites examined. In one further feed (MF59), six of seven infected midguts carried *pfmdr1*-184F and one had *pfmdr1*-184Y. In all other cases where multiple oocyst-positive midguts arose from a single feed, they contained identical parasite genotypes (Table S1).

The most infectious gametocyte sample (MF68) originated from a CQ/SP-treated patient, producing 13 infected midguts with a mean of 42 oocysts on each (range, 2–100). Eleven of the 13 midguts were PCR-positive for all four resistance-associated genes. CQR alleles *pfcrt*-76T, *pfmdr1*-86Y, and *pfmdr1*-184F were detected pre- and posttreatment and in all 11 midguts. The *pfdhfr* genotype was mixed with both wild-type and resistance-associated alleles detected at positions 51, 59, and 108 both pre- and posttreatment. However, none of these wild-type alleles were detected in the resulting 11 midguts, all of which harboured pure *pfdhfr* triple mutants (51-I, 59-R, and 108-N).

### Do Resistant Infections at Day 0 Contribute More to Later Transmission?

Very few changes in parasite genotype at resistance-associated loci were observed from pretreatment sampling to identification of markers in infected mosquito midguts. Therefore, we used day 0 data to look for associations between multidrug-resistant genotypes and mean oocyst count in mosquitoes membrane-fed on posttreatment emergent gametocytes (i.e., considering only those subjects without gametocytes at time of treatment). The presence of parasites with the four-locus multidrug-resistant genotype TYRG at day 0 was associated with higher oocyst numbers in SP and CQ/SP groups, but this was not statistically significant in either group (for SP, *N* = 644 dissected mosquitoes: mean ratio, 4.14; 95% CI, 0.722–23.8; *p* = 0.111; for CQ/SP, *N* = 415: mean ratio, 5.53; 95% CI, 0.742–41.2; *p* = 0.095). In contrast, in the CQ group, only one donor harboured TYRG parasites at day 0, and there was no transmission from this child's gametocytes. Parasites carrying CQ resistance markers at *pfcrt* and *pfmdr1* without accompanying antifolate resistance mutations in *pfdhfr* and *pfdhps* were highly transmissible in the CQ group (*N* = 186; mean oocyst ratio: 261; 95% CI, 24.8–2736; *p* < 0.001). This is consistent with previous findings [13].

### Gametocyte and Oocyst Genotypes Impact Significantly on Transmission Success

An unambiguous four-locus genotype could be assigned to gametocyte feed samples from 56 children. Where mixed infections were detected, these were scored as resistant. Fourteen of the 56 were from children who had harboured gametocytes at the time of treatment; four, eight, and two children from the CQ, SP and CQ/SP treatment groups, respectively. We analysed the contribution of TYRG parasites to transmission in the 1680 mosquitoes that had fed on these 56 gametocyte samples, after stratifying by drug group. This was carried out by calculating the mean oocyst number on midguts resulting from TYRG-positive feed samples and comparing this to the mean oocyst number on midguts resulting from feeds containing any other genotypes, and producing a ratio of the means. In the same way, we then compared oocyst burden in the 48 midguts for which four-locus genotype data were available. Results of both analyses are presented in Table 3.

**Table 3:**
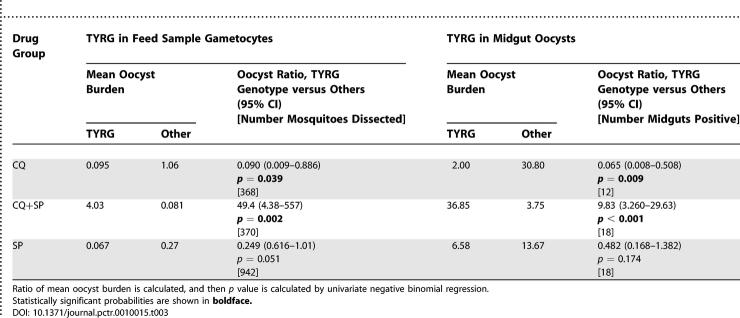
Contribution to Transmission Success of Multilocus Resistant Genotypes in Gametocytes and Oocysts

The presence of TYRG in gametocyte samples and in oocyst DNA was associated with a significantly lower oocyst burden from CQ-treated individuals. In the SP-treated group, TYRG gametocytes also led to lower oocyst burdens, with borderline significance (*p* = 0.051). However, after CQ/SP treatment, parasites carrying TYRG resulted in significantly greater transmission success compared to other genotypes in this treatment group.

## DISCUSSION

### Interpretation

When compared to the rapidly waning efficacy of CQ monotherapy in our study area [7], addition of SP to CQ provided therapeutic efficacy of over 90% after PCR correction in both this trial in 2001, and in a trial carried out 1 y later [11,15]. CQ/SP has been the recommended treatment for uncomplicated malaria in the Gambia since 2004. SP alone has had a measured parasitological efficacy of approximately 90% over 28 d in the Gambia [15,19]. However, both SP and CQ/SP are characterized by substantial rates of posttreatment gametocyte emergence [9,11], and because alleles of *pfdhfr* and *pfdhps* associated with SP failure are present in the Farafenni area, we expected that parasites resistant to both drugs would be frequently transmitted following combination treatment.

In this study, CQ/SP treatment was followed by little reduction in posttreatment prevalence and density of gametocytes, compared to CQ alone. Only at the day 28 follow-up point was gametocyte carriage in the CQ/SP-treated group significantly lower than in the CQ group. However, gametocytes occurred more frequently and at higher density in the SP group throughout posttreatment follow-up and particularly at day 28 (Figure 3), suggesting that CQ/SP-treated children may be infectious for a shorter period of time following treatment than those treated with SP alone. This may be the result of more rapid asexual parasite clearance by CQ/SP [8], combined with the activity of CQ against early developing gametocytes [6].

Gametocyte carriers from all three treatment groups were infectious to A. gambiae in experimental feeding. Remarkably, the high gametocyte densities observed among the SP-treated group at day 7 in particular did not translate into substantial infectiousness (Figure 3). Gametocyte carriers in the SP group identified at day 10 or 14 were significantly more infectious to mosquitoes, suggesting that gametocytes circulating at day 7 after SP treatment, but not CQ or CQ/SP treatment, have not attained sufficient maturity to infect mosquitoes efficiently. One explanation for this observation is that, because SP appears unable to kill even the earliest developing gametocyte stages sequestered in the host tissues at the time of treatment [9], many of the emergent gametocytes seen on day 7 are not as yet fully mature, although morphologically they appear so. In contrast, CQ will kill genotypically sensitive gametocytes in the early stages of development, and so day 7 emergent gametocytes are those that were already some way to maturity at the time of treatment. However, this does not explain why the significant numbers of CQ-resistant parasites were not also less infectious at day 7 in the CQ treatment group, because their early development is expected to be unimpaired by drug. An alternative hypothesis is that SP treatment in some way perturbs the gametocyte sex ratio, so that the accumulation of one sex is delayed, thus preventing fertilization until sufficient of both sexes are present in the gametocyte population. It remains unclear why the maturity of gametocytes was a particularly important factor in the infectiousness of children who had also harboured gametocytes at day 0.

Resistance-associated alleles at five of the seven loci examined were more common among gametocyte carriers than among noncarriers in a randomly selected group of pretreatment isolates, as was the *pfdhfr* “triple mutant” haplotype of 51-I, 59-R, and 108-N (Figure 4). The multilocus genotype TYRG (*pfcrt-*76T, *pfmdr1*-86Y, *pfdhfr*-59R, and *pfdhps-*437G) was also more common among gametocyte carriers, and among children donating gametocytes for feeding experiments, than among noncarriers, although this was not statistically significant. In the accompanying paper, the TYRG genotype was found to be weakly associated with parasitological failure after SP treatment, but not after CQ/SP treatment [15].

The presence of the TYRG multilocus genotype among either gametocytes membrane-fed to mosquitoes, or among P. falciparum oocysts present in infected mosquito midguts was associated with significantly higher oocyst burdens in the CQ/SP combination group. This was independent of feed type or the presence of gametocytes prior to treatment, and was much stronger than the association between this genotype and treatment failure [15]. TYRG carrying parasites thus appear to enjoy a substantial survival advantage, which leads to favourable transmission under CQ/SP treatment. In contrast, TYRG genotypes were less successfully transmitted than other genotypes in both monotherapy arms; in the CQ treatment group, this difference was statistically significant. This supports the view that drug-resistant parasites are likely to be less fit, relative to other genotypes in the population, in the absence of the particular drug or combination of drugs that has led to their selection [20].

### Generalizability

Our data did not provide clear evidence of within-host selection for the TYRG genotype, as we have previously observed in CQ-treated patients [12,13]. This is probably due to the fact that some of the alleles, particularly *pfcrt-*76T and *pfdhfr-*108N, were at a high starting prevalence and so wild-type alleles were rare.

Taken together, our analyses of therapeutic and transmission endpoints of this clinical trial demonstrate that multidrug-resistant parasites enjoyed a survival advantage in terms of their likelihood of transmission to mosquitoes, but that the particular genotypes encountered in the Gambia did not threaten the therapeutic efficacy of CQ/SP [11,15]. Pretreatment genotyping of 90 children in a subsequent trial in 2002 [11] showed that *pfdhfr* and *pfdhps* mutation prevalences remained stable (*pfdhfr* triple mutant present in 50 of 89 or 56.2%; 95% CI, 45.9**–**66.5; *pfdhps* 437G present in 39 of 79 or 49.4%; 95% CI, 38.4**–**60.4) (R. Hallett and C. Sutherland, unpublished data). However, CQ monotherapy remained first line treatment at this time and CQ/SP was not being commonly used. The apparent anomaly between the widespread prevalence of the *pfdhfr* triple mutant in Africa and its weak association with risk of treatment failure [21] may simply be because its primary effect has been to enhance transmission of parasites under SP drug pressure, and this has been sufficient to ensure its persistence and spread.

### Overall Evidence

The work presented in this paper extends our previous studies of CQ-treated, SP-treated, and CQ/SP-treated patients in this study area [9–11,13]. There are no previous published studies to our knowledge that measure transmission endpoints among African patients treated with CQ/SP. We have shown that multidrug-resistant P. falciparum are prevalent in West Africa and that transmission of these genotypes is favoured when CQ and SP are combined for treatment of uncomplicated malaria in children. Thus, evolution of resistance to this combination will continue despite any reduction in its therapeutic efficacy, so far. These findings demonstrate the importance of measuring transmission endpoints in studies of the evolution of resistance to antimalarial chemotherapy.

## SUPPORTING INFORMATION

CONSORT Checklist Click here for additional data file. (50 KB DOC)

Trial Protocol Click here for additional data file. (88 KB DOC)

Table S1 (343 KB DOC)

## Abbreviations

ACT: artemisinin-containing combination therapy
CI: confidence interval
CQ: chloroquine
SP: sulphadoxine-pyrimethamine.

## References

[pctr-0010015-b001] Brockman A, Price RN, van Vugt M, Heppner DG, Walsh D (2000). Plasmodium falciparum antimalarial drug susceptibility on the north-western border of Thailand during five years of extensive use of artesunate-mefloquine. Trans R Soc Trop Med Hyg.

[pctr-0010015-b002] Meerman L, Ord R, Teun Bousema J, van Niekerk M, Osman E (2005). Carriage of chloroquine-resistant parasites and delay of effective treatment increase the risk of severe malaria in Gambian children. J Infect Dis.

[pctr-0010015-b003] Roper C, Pearce R, Nair S, Sharp B, Nosten F (2004). Intercontinental spread of pyrimethamine-resistant malaria. Science.

[pctr-0010015-b004] Bell D, Winstanley P (2004). Current issues in the treatment of uncomplicated malaria in Africa. Br Med Bull.

[pctr-0010015-b005] Price RN, Nosten F, Luxemburger C, ter Kuile FO, Paiphun L (1996). Effects of artemisinin derivatives on malaria transmissibility. Lancet.

[pctr-0010015-b006] Butcher GA (1997). Antimalarial drugs and the mosquito transmission of *Plasmodium*. Int J Parasitol.

[pctr-0010015-b007] Sutherland CJ, Drakeley CJ, Obisike U, Coleman R, Jawara M (2003). The addition of artesunate to chloroquine for treatment of Plasmodium falciparum malaria in Gambian children delays, but does not prevent treatment failure. Am J Trop Med Hyg.

[pctr-0010015-b008] Bojang KA, Schneider G, Forck S, Obaro SK, Jaffar S (1998). A trial of Fansidar^®^ plus chloroquine or Fansidar^®^ alone for the treatment of uncomplicated malaria in Gambian children. Trans R Soc Trop Med Hyg.

[pctr-0010015-b009] Targett GAT, Drakeley CJ, Jawara M, von Seidlein L, Coleman R (2001). Artesunate reduces but does not prevent posttreatment transmission of Plasmodium falciparum to Anopheles gambiae. J Infect Dis.

[pctr-0010015-b010] Drakeley CJ, Jawara M, Targett GA, Walraven G, Obisike U (2004). Addition of artesunate to chloroquine for treatment of Plasmodium falciparum malaria in Gambian children causes a significant but short-lived reduction in infectiousness for mosquitoes. Trop Med Int Health.

[pctr-0010015-b011] Sutherland CJ, Ord R, Dunyo S, Jawara M, Drakeley CJ (2005). Reduction of malaria transmission to anopheles mosquitoes with a six-dose regimen of co-artemether. PLoS Med.

[pctr-0010015-b012] Sutherland CJ, Alloueche A, Curtis J, Drakeley CJ, Ord R (2002). Gambian children successfully treated with chloroquine can harbour and transmit Plasmodium falciparum gametocytes carrying resistance genes. Am J Trop Med Hyg.

[pctr-0010015-b013] Hallett RL, Sutherland CJ, Alexander N, Ord R, Jawara M (2004). Combination therapy counteracts the enhanced transmission of drug-resistant malaria parasites to mosquitoes. Antimicrob Agents Chemother.

[pctr-0010015-b014] Méndez F, Muňoz Á, Carrasquilla G, Jurado D, Arévalo-Herrera M (2002). Determinants of treatment response to sulphadoxine-pyrimethamine and subsequent transmission potential in falciparum malaria. Am J Epidemiol.

[pctr-0010015-b015] Dunyo S, Ord R, Hallett R, Jawara M, Walraven G (2006). Randomised trial of chloroquine/sulphadoxine-pyrimethamine in Gambian children with malaria: Impact against multidrug-resistant Plasmodium falciparum. PLoS Clin Trials.

[pctr-0010015-b016] Plowe CV, Djimde A, Bouare M, Doumbo O, Wellems TE (1995). Pyrimethamine and proguanil resistance-conferring mutations in Plasmodium falciparum dihydrofolate reductase: polymerase chain reaction methods for surveillance in Africa. Am J Trop Med Hyg.

[pctr-0010015-b017] Walsh PS, Metzger DA, Higuchi R (1991). Chelex^®^ 100 as a medium for simple extraction of DNA for PCR-based typing from forensic material. BioTechniques.

[pctr-0010015-b018] Pearce RJ, Drakeley C, Chandramohan D, Mosha F, Roper C (2003). Molecular determination of point mutation haplotypes in the dihydrofolate reductase and dihydropteroate synthase of Plasmodium falciparum in three districts of northern Tanzania. Antimicrob Agent Chemother.

[pctr-0010015-b019] von Seidlein L, Milligan P, Pinder M, Bojang K, Anyalebechi C (2000). Efficacy of artesunate plus pyrimethamine-sulphadoxine for uncomplicated malaria in Gambian children: a double-blind, randomised, controlled trial. Lancet.

[pctr-0010015-b020] Hayward R, Saliba KJ, Kirk K (2005). *pfmdr1* mutations associated with chloroquine resistance incur a fitness cost in Plasmodium falciparum. Mol Microbiol.

[pctr-0010015-b021] Dorsey G, Dokomajilar C, Kiggundu M, Staedke SG, Kamya MR (2004). Principal role of dihydropteroate synthase mutations in mediating resistance to sulfadoxine-pyrimethamine in single-drug and combination therapy of uncomplicated malaria in Uganda. Am J Trop Med Hyg.

