# Supplementary material for: Chloroquine/Sulphadoxine-Pyrimethamine for Gambian Children with Malaria: Transmission to Mosquitoes of Multidrug-Resistant Plasmodium falciparum
Source: PLoS Clin Trials. 2006 Jul 21;1(3):e15. doi: 10.1371/journal.pctr.0010015 (PMC1513405; doi:10.1371/journal.pctr.0010015)
Supplement: Trial Protocol [file pctr.0010015.sd002.doc]

# Medical Research Council Laboratories, Fajara

**Application to undertake a research project**

**__________________________________________________________________________**

#### A Summary Information

**A1 Title of project**

Please choose a short clear title for ease of reference and identification in future.

The impact of anti-malarial treatment upon the development and persistence of *Plasmodium falciparum* gametocytes *in vitro* an*d in vivo.*

**A2 Investigators (Principal Investigator first)**

Please list all investigators and collaborators and attach CV if the principal investigator is unknown to the Committee

| **Name** | Institution | Position |
| --- | --- | --- |
| Sutherland, Colin J. | London School of Hygiene and Tropical Medicine Medicine | Research Fellow |
| Targett, Geoffrey A.T. | LSHTM | Professor |
| Milligan, Paul | MRC Laboratories, Fajara | Statistician |
| Walraven, Gijs | MRC Field Station, Farafenni | Field Station Head |
| Juwara, Musa | MRC Field Station, Farafenni | Entomologist |
| Dunyo, Sam | MRC Field Station, Farafenni | Clinical Epidemiologist |
| Pinder, Margaret | MRC Laboratories, Fajara | Programme Head |
|  |  |  |

**Who will introduce the proposal at SCC?**

Dr Margaret Pinder

**A3 Location(s) of research**

Please list all the places where the research will take place including field sites or health facilities

MRC Field Station, Farafenni

Farafenni AFPRC Hospital/MCH clinic

London School of Hygiene and Tropical Medicine

**A4 Proposed start date and duration in months**

In giving a date please bear in mind the timescale for decision-making by SCC, the Ethical Committee and any other institutions whose agreement is needed, and the time needed to organise the resources required.

September 2001, for 3.5 months at Farafenni; data analysis to follow in London and The Gambia.

NB. This study follows that of 2000 by the same investigators, in which we measured post-treatment transmission among children treated with chloroquine plus artesunate.

**A5 Reference (office use)**

**Scientific Co-ordinating Committee No Project #887**

**Ethical Committee No #887**

**A6 Summary of project, long term objectives and specific aims (not more than 200 words)**

This section is very helpful to the Committees in determining quickly the main features of the study, and should be as clear and concise as possible. It should cover the key objectives and endpoints and, if the project is hypothesis driven, then the hypothesis should be stated here.

Widespread resistance of *Plasmodium falciparum* to commonly-used drugs has lead to the formulation and testing of new combinations of anti-malarials, particularly those including one of the artemisinins. This proposal is one in a series of trials aimed at identifying drug combinations that reduce or prevent post-treatment transmission of *P. falciparum*. Our studies in 1998/9 have shown that:

- Sulphadoxine/pyrimethamine (SP; Fansidar®) treatment is followed by high gametocytaemia in a large proportion of subjects.
- Compared to SP, artesunate + SP reduces, but does not prevent, transmission of *P. falciparum* to mosquitoes.

Further, preliminary analysis of our 2000 trial suggests that:

- the prevalence of resistance to chloroquine is sufficiently high in The Gambia to severely compromise chloroquine + artesunate as a treatment for uncomplicated malaria.
- chloroquine + artesunate reduces transmission at day 7, but this benefit is probably transient, as gametocyte carriage increases significantly in the latter half of follow-up.

Thus whereas both of the artesunate combinations we have tested do reduce the infectiousness of treated children, neither can completely prevent transmission. It would be very useful to know whether the commonly used combination of chloroquine and sulphadoxine-pyrimethamine (SP, Fansidar) can also reduce transmission. We propose to test the infectiousness of children treated with chloroquine and SP as in The Gambia this combination has been shown to be a more effective treatment for symptomatic malaria than SP alone (13) and is currently the recommended treatment for complicated malaria (14). However gametocyte prevalence, density and transmissibility to mosquitoes have not been systematically measured in patients treated with chloroquine and SP. We would also include treatment arms of both PS and CQ alone.

This protocol fits well with the overall research aims of our programme of research, and has the added benefit that the drugs concerned are low cost and already available in The Gambia. This combination may thus be considered as a possible alternative to the current front-line therapy of CQ alone.

#### A7 Checklist/Signatures

**Please complete the following checklist and comment as appropriate.** This section is designed to ensure that all the planning steps have been taken that are needed for successful project. For projects at the MRC Laboratories, Programme Heads will help visiting workers, and others preparing proposals at a distance, to ensure liaison with key individuals who need to be consulted locally.

1. **Has the project been discussed and cleared with the institutions in which research will be carried out including health services to which the study will need access?**

Yes

1. **Have all investigators and collaborators given their agreement to take part in the study as described?**

Yes

1. **Has a CV been attached for the principal investigator if unknown to the Committee?**

Not applicable

1. **Have ethical issues been addressed? Give details in section C.**

Yes

1. **Have safety issues been addressed? Please give details**

Yes

1. **Will the project require data and/or materials to be taken out of The Gambia? If so please give details** Data will be analysed at LSHTM in collaboration with MRC staff in The Gambia. We plan to take the following material out of The Gambia to either LSHTM or the University of Edinburgh (*):

- filter-paper blood spots for genotyping studies
- small (~50μl) blood samples for RNA extraction *
- alcohol-fixed oocysts from mosquito guts
- frozen sera used in transmission experiments
- paraformaldehyde-fixed parasites on microscops slides for immunofluoresence*

1. **For projects to be carried out at MRC Laboratories: Have the following been consulted about the support services, resources and working space required?**

|  | **Consulted: Yes/No/Comment** |
| --- | --- |
| Laboratory Manager (including safety issues) | Ongoing |
| Director of Clinical Services | Project at AFPRC hospital/MCH clinic, discussed with CE hospital, and DHT |
| Head of Computing | Data-entry at Farafenni field station, overseen by Maimuna Sowe |
| Transport Manager | Ongoing |
| Finance Manager | Ongoing |
| Personnel Manager | Not available |
| Administrative Director | Not available |
| Other services – specify | Modest requirement for printing forms to be done at Farafenni |

**Signature of principal investigator: Date:**

**__________________________________________________________________________**

**B Description of Project**

**B1 Background**

In response to the widespread occurrence of chloroquine-resistant parasites in Africa, and the more recent demonstrations that resistance to sulphadoxine/pyrimethamine (SP) is reducing the efficacy of that drug (6,8), calls have been made for the deployment of new effective antimalarial combinations. Of particular interest are the artemisinins, which have short elimination half-lives, in various combinations with longer-acting drugs (11,12). Recent trials of artesunate in combination with SP in Africa have shown this to be a safe and efficacious regimen (9,10), but that a certain level of post-treatment transmission occurs (7,10). It is of importance to determine the effects of the artemisinins on *P. falciparum* transmission, and to use this information in the formulation of appropriate combination therapies for use in Africa.

We have run a series of trials in which we can measure *P. falciparum* gametocyte prevalence, density and infectivity to mosquitoes after treatment with artemisinins in various combinations with other anti-malarials. This work has been carried out at the MRC field station at Farafenni, The Gambia, during three consecutive malaria transmission seasons (1998 and 1999; ref. 7; 2000, Sutherland et al., unpublished). In total 1,636 children have been treated with either chloroquine alone, chloroquine plus artesunate, SP alone, or SP plus artesunate.

## Our studies in 1998/9 showed that artesunate, in combination with SP, reduced both the mean density of gametocytes in positive subjects, and the prevalence of gametocytes in the treated population (7) compared to SP alone. Nevertheless, a gametocyte prevalence of 5-10% was observed at day 7 in the combination group, and these gametocytes were capable of infecting mosquitoes in membrane-feeding experiments (7). Therefore, there may still be considerable transmission after treatment with artesunate combined with SP.

In 2000, we compared treatment with the combination chloroquine plus artesunate (3 days) with chloroquine alone. We have not completed analysis of this trial yet, as a duplicate slide-read is currently ongoing, but the following summary of our preliminary findings can be made:

- parasitological treatment failures with chloroquine alone were very common, the cumulative rate at day 28 approaching 60% (although this is a crude figure which includes re-infections)
- parasitological treatment failures with chloroquine plus artesunate were also unacceptably high, with a cumulative rate at day 28 of 39%, although on average these occurred much later in follow-up that failures with chloroquine alone.
- Clinical failures mirrored this pattern, with cumulative failure rates by day 28 of 12% and 8% respectively. The addition of artesunate to chloroquine treatment significantly reduced the probability of a child returning with clinical malaria within 14 days of treatment, but this benefit was not observed in the latter half of follow-up.
- Gametocyte carriage was significantly reduced in the combination group up to day 14, but day 28 gametocyte carriage rates were high in both groups: 34% for chloroquine and 21% for chloroquine +artesunate.
- Children identified as gametocyte carriers on day 7, but who were gametocyte-negative on admission, were tested for infectiousness to *Anopheles*. Some gametocyte-carriers from both groups were infectious (12/26 for the chloroquine group; 4/17 for the combination group), although gametocytes from children receiving chloroquine plus artesunate were less infective.

We conclude from this preliminary analysis that whereas addition of artesunate to chloroquine does reduce transmission immediately post-treatment, the emergence of asexual and sexual parasites in the 3^rd^ and 4^th^ weeks greatly reduces this benefit over the longer term.

Since none of the combinations so far tested are ideal to reduce transmission and it is important to know if they are superior to the commonly used combination of chloroquine and sulphadoxine-pyrimethamine (SP, Fansidar). We propose to test the infectiousness of children treated with chloroquine and SP as in The Gambia this combination has been shown to be a more effective symptomatic treatment than SP alone for malaria (13) and is currently the recommended treatment for complicated malaria (14). However gametocyte prevalence, density and transmissibility to mosquitoes have not been systematically measured in patients treated with chloroquine and SP. We would compare the results with those obtained among groups of children treated with CQ alone and with SP alone.

This protocol fits well with the overall research aims of our programme of research, and has the added benefit that the drugs concerned are low cost and already available in The Gambia. This combination may thus be considered as a possible alternative to the current front-line therapy of CQ alone.

In all our studies to date, we have examined gametocyte infectivity by feeding mosquitoes either 4 or 7 days after treatment. Our parasitological data show that gametocytes persist, particularly after SP treatment, for up to 3 weeks beyond this point (7). These persistent circulating gametocytes may no longer be fully viable. However, if they are infective they could represent a significant source of further transmission, due to the lengthy time period during which they are available for mosquitoes to ingest them. The Wellcome Trust grant review panel strongly suggested that this question be incorporated into our study design within the period of the grant. For this reason, we intend to stagger the gametocyte screening process among those children that receive SP to examine the relative infectivity of gametocytes isolated 7, 10 or 14 days following treatment.

### B2 Project description

Primary aim:

- To measure *P.falciparum* transmission after chloroquine and SP vs either treatment alone.

Secondary aims:

- To measure the duration of infectivity of gametocytes isolated from SP-treated children.
- To measure the genetic complexity of gametocyte populations
- To measure the prevalence of alleles associated with drug resistance

**Specific objectives:**

1. Using a randomised control trial (RCT) design, measure the transmission potential of Gambian children after treatment with anti-malarial drugs alone and in combination. Using gametocyte prevalence, density and infectivity to mosquitoes as outcome measures, we plan to test whether chloroquine and SP effectively reduces transmission compared to either treatment alone and, by comparison with our previous work, compared to combinations including artesunate. To do this, we will set up a randomised control trial of chloroquine and PS versus groups treated with either PS or chloroquine.
2. Measure infectivity in a staggered group of gametocyte isolates, collected at day 7, day 10, and day 14 after SP treatment. This is because our studies to date have not enabled us to estimate the duration of infectivity of gametocytes that appear after drug treatment.
3. Examine genetic complexity of gametocyte populations *in vivo* by PCR analysis of polymorphic loci, and use RT-PCR of peripheral blood RNA to establish stage specificity of parasite samples. We will further develop these techniques in collaboration with H. Babiker and D. Walliker (1).
4. Use PCR genotyping at putative drug-resistance loci, in combination with data on treatment failures, to estimate the prevalence of resistant parasites in the emerging gametocyte population. Oocysts from infected mosquitoes will also be typed. Quantification of the relative contribution of different genotypes to circulating gametocyte pools will be estimated using the *in situ* PCR approach recently developed by our collaborators L. Ranford-Cartwright and D. Walliker (5).

**Experimental Design and Methods to be Used**

###### Clinical trial and transmission experiments

Over the three previous seasons, we have established a randomised control trial (RCT) protocol, modified from the standard WHO guidelines for efficacy trials, for the examination of post-treatment transmission of falciparum malaria.

Children aged 1-10 years attending the Farafenni AFPRC Hospital and MCH clinic requiring treatment for uncomplicated malaria will be recruited into the study after appropriate consent has been obtained from a parent or guardian. They will be randomly assigned to a treatment group by clinicians of the MRC Station, Farafenni. Clinical and demographic data will be recorded on a standardised case report form (CRF) as in previous years. The principal investigator and entomologists will be blinded as to the treatment group of each child. Field workers will be required to monitor drug doses on days 2 and 3 in the child’s home and therefore cannot be blinded. Blood films, blood spots and a microtainer blood sample will be obtained at the time of treatment. The blood sample will be used to measure PCV, genotyping parasites and previous chloroquine intake.

All subjects will be actively followed up at home for two weeks on either days 7 or 10, and on days 14 and 28 post-treatment. Parents and guardians will be encouraged to bring the child back to the clinic at any other time the child is unwell. On one follow-up day (7, 10 or 14) the child and a guardian will be brought to the field station for gametocyte screening. On each occasion, 2 thick films will be taken, and blood spotted onto filter paper for genotyping. On the gametocyte screening day, the child will be asked to remain at the field station until one thick film has been read. If gametocytes are present, and permission given, 2 to 3 ml of blood will be taken by venupuncture and immediately processed for mosquito infection studies.

Farafenni has two purpose-built insectaries. We plan to use this protocol for the next two consecutive transmission seasons at Farafenni for comparative studies of post-treatment malaria transmission. Currently we are breeding F1 generation *Anopheles gambiae* from wild caught mosquitoes for these experiments but it is a priority in the coming season is to establish a stable colony of *A. gambiae* in the Farafenni insectaries.

In our 1998, 1999 and 2000 trials based at the Farafenni Health Centre we recruited 600 children over 15 weeks, 500 children over 10 weeks, and 536 children over 9 weeks respectively. These rates of subject recruitment, combined with gametocyte prevalence data from each year will be fed into the study design to ensure sufficient mosquito feeds across the treatment groups.

We will analyse the results of the trial for all the relevant clinical, parasitological and entomological parameters available to us. In addition, we have used two summary statistics (the probability of post-treatment infectiousness, and the infectious proportion of subjects) that incorporate gametocyte prevalence, density and infectivity to mosquitoes (7). These data will then be related to the estimated proportion of treated infections in rural areas of The Gambia to derive an estimate of the contribution that children treated with each regimen would make to transmission.

###### Parasite genotyping

We intend to establish the necessary capacity to perform basic molecular biology in the laboratories at Farafenni. Along with our collaborators at Edinburgh University, we have secured funding for a PCR machine and electrophoresis equipment from the Gates Malaria Programme at LSHTM, and the MRC. We have also obtained sufficient funding to support a higher scientific officer with experience in molecular biology. She/he will be trained in the specific techniques used in these studies and be responsible for these aspects of the work in the field station.

We propose to measure the genetic complexity of gametocyte populations in individuals, and to relate this to the genetic complexity of oocysts in mosquitoes fed on those individuals. This will permit us to track longitudinally the movement of drug-resistant alleles from asexual parasites, to gametocytes, to mosquitoes. From blood spotted on filter paper, we have been able to use PCR genotyping of the polymorphic single locus genes encoding MSP1, MSP2 and GLURP to elucidate the genetic complexity of circulating gametocytes. We prefer to use antigen-encoding loci for this purpose rather than “neutral” markers such as micro-satellites because of their greater within-population diversity, which provides better resolution among genotypes.

A novel application of RT-PCR has allowed us to detect expression of developmental stage-specific genes in peripheral blood obtained at the time of feeding mosquitoes (Sutherland *et al.,* in preparation). RT-PCR of an asexual-specific locus (*resa*) and a gametocyte-specific locus (*pfs16*) permits samples with gametocytes only and samples with sub-patent asexual parasites as well to be distinguished from each other. Thus we can clarify whether the DNA in our genotyping experiments is derived from gametocytes only, or from a combination of gametocytes and asexual parasites at the limit of detection of our RT-PCR system. These techniques are complementary to those of our collaborators H. Babiker and D. Walliker (1). The results of this molecular genetics approach have clearly demonstrated that day 4 and day 7 circulating gametocytes comprise a mix of, on average, two or more genotypes. Thus in our study area the requirements are being met for heterologous genetic recombination to occur in the mosquito. This is the first time such an analysis has been reported. However, this work included only 10% of our study subjects, and a meaningful comparison among treatment groups was not possible with this sample size. We plan an expansion of this work to other drug treatment groups, and with larger sample sizes, using these methodologies.

We propose to determine the genotype of our samples at loci involved in drug resistance. We will type at both the *dhfr* and *dhps* loci implicated in resistance to SP. These analyses will provide crucial information as to the contribution of drug-resistant loci to the emerging gametocyte pool, and thus give much-needed empirical data concerning the rate of increase of the prevalence of drug resistant genotypes in African parasite populations. Further, using the combination of chemotherapy trials with mosquito infectivity studies, we will be able to follow drug-resistant genotypes into the mosquito, by dissecting oocysts from infected mosquitoes and typing them with established PCR methods (2-4). Thus we will have longitudinal genetic typing data from 3 life-cycle compartments originating from a single infection: asexual parasites (taken at the time of clinical presentation), gametocytes (taken at day 7 after treatment has largely removed the asexual parasites) and oocysts from the mosquito.

Using DNA prepared from filter paper blood spots we will use PCR to amplify candidate loci, particularly *pfcrt*, *pfmdr1*, *dhfr* and *dhps*. These results will be correlated with both clinical data (i.e. which subjects failed treatment), and parasitological data (i.e. which subjects had persisting patent asexual parasitaemias after treatment). This will enable us to build up a comprehensive picture of the prevalence of drug resistance alleles in emerging gametocyte populations in the field after drug treatment. The technique of *in situ* PCR, to be performed in collaboration with Prof. David Walliker and Lisa Ranford-Cartwright, will enable us to evaluate the relative abundance of gametocytes carrying drug-resistance loci in our gametocyte samples prior to feeding to mosquitoes. The subsequent typing of single oocysts from mosquitoes successfully infected with those same gametocytes will then allow us to evaluate the post-transmission abundance of these same loci.

1. Babiker, H. A., Abdel-Wahab, A., Ahmed, S., Suleiman, S., Ranford-Cartwright, L.C., Carter, R. and Walliker, D. 1999. Detection of low level *Plasmodium falciparum* gametocytes using reverse transcriptase polymerase chain reaction. *Mol. Biochem. Parasitol.* **99:** 143-148.
2. Babiker, H.A., Ranford-Cartwright, L.C., Currie, D., Charlwood, J.D., Billingsley, P., Teuscher, T. and Walliker, D. 1994. Random mating in a natural population of the malaria parasite *Plasmodium falciparum*. Parasitology **109:** 413-421.
3. Ranford-Cartwright, L.C., Balfe, P., Carter, R. and Walliker, D. 1991. Genetic hybrids of *Plasmodium falciparum* identified by amplification of genomic DNA from single oocysts. *Mol. Biochem. Parasitol.* **49:** 239-244.
4. Ranford-Cartwright, L.C., Balfe, P., Carter, R. and Walliker, D. 1993. Frequency of cross-fertilization in the human malaria parasite *Plasmodium falciparum*. *Parasitology* 1**07:** 11-18.
5. Ranford-Cartwright, L.C., and Walliker, D. 1999. Intragenic recombinants of *Plasmodium falciparum* identified by in situ polymerase chain reaction *Mol. Biochem. Parasitol.* **102:** 13-20.
6. Ronn, A.M., Msangeni, H.A., Mhina J., Wernsdorfer W.H., Bygbjerg I.C. 1996. High level of resistance of *Plasmodium falciparum* to sulfadoxine-pyrimethamine in children in Tanzania. *Trans R Soc Trop Med Hyg*. **90:** 179-81.
7. Targett, G.A.T., Drakeley, C.J., Jawara, M., von Seidlein, L., Coleman, R., Deen, J., Pinder, M., Doherty, T., Sutherland, C., Walraven, G., and Milligan, P. 2000. The effects on transmission of *Plasmodium falciparum* malaria of treatment with pyrimethamine/ sulphadoxine alone or in combination with artesunate. 2001; 183: 1254-1259
8. Trigg JK, Mbwana H, Chambo O, Hills E, Watkins W, Curtis CF. 1997. Resistance to pyrimethamine/sulfadoxine in *Plasmodium falciparum* in 12 villages in north east Tanzania and a test of chlorproguanil/dapsone. *Acta Trop* **63:** 185-189.
9. von Seidlein L, Milligan P, Pinder M, Bojang K, Anyalebechi C, Gosling R, Coleman R, Ude JI, Sadiq A, Duraisingh M, Warhurst D, Alloueche A, Targett G, McAdam K, Greenwood B, Walraven G, Olliaro P, Doherty T. 2000. Efficacy of artesunate plus pyrimethamine-sulphadoxine for uncomplicated malaria in Gambianchildren: a double-blind, randomised, controlled trial.*Lancet*. **355:** 352-7.
10. von Seidlein, L., Bojang, K., Jones, P., Jaffar, S., Pinder, M., Obaro, S., Doherty, T., Haywood, M., Snounou, G., Gemperli, B., Gathmann, I., Royce, C., McAdam, K., and Greenwood, B. 1998. A randomized controlled trial of artemether/benflumetol, a new antimalarial and pyrimethamine/sulfadoxine in the treatment of uncomplicated falciparum malaria in African children *Am. J. Trop. Med. Hyg.* **58:** 638-644.
11. Watkins, W.M. and Mosobo, M. 1993. Treatment of *Plasmodium flciparum* malria with pyrimethamine-sulfadoxine: selective pressure for resistance is a function of long elimination half-life. *Trans. Roy. Soc. Trop. Med. Hyg.* **87:** 75-78.
12. White NJ, Nosten F, Looareesuwan S, Watkins WM, Marsh K, Snow RW, Kokwaro G, Ouma J, Hien TT, Molyneux ME, Taylor TE, Newbold CI, Ruebush TK 2nd, Danis M, Greenwood BM, Anderson RM, Olliaro P. 1999. Averting a malaria disaster. *Lancet* **353:** 1965-1967
13. Bojang KA, Schneider G, Forck S, obaro SK, Jaffar S, Pinder M, Rowley J, Greenwood BM. A trial of Fansidar® plus chloroquine or Fansidar® alone for the treatment of uncomplicated malaria in Gambian children**. *Trans R Soc Trop Med Hyg* 1998;** 92**: 73-76.**
14. Standard drug treatment guidelines. 1998 Dept for Health and Women’s Affairs, The Gambia

**B3 Details of study design and investigations**

1. **What type of study design is proposed (eg case control, prospective cohort, randomised controlled trial, descriptive etc)**

Randomised Control Trial

1. **What is the proposed size of the study (this may relate to patients, cases, controls, survey subjects, laboratory samples etc, as appropriate).**

The aim is to recruit approximately 500 patients, to have a minimum of 25 individuals per group who provide a blood sample for membrane feeding.

1. **Please describe the statistical considerations and sample size calculations involved in determining the size of the study.** (If you do not have access to statistical advice, please consult the MRC Laboratories Statistics Department.)

Approximately 500 patients will be treated. In our previous studies the observed gametocyte carriage rate 7 days after treatment with SP was >70% (excluding those who had gametocytes on day 0), and 40% after treatment with CQ. We therefore expect that the rate after SP + CQ will be at least 28% (ie 40% x 70%). Therefore, to ensure that at least 30 mosquito feeds are performed in each treatment group, we will aim to enrol **a)** 200 subjects in the CQ + SP group, **b)** 50, 60 and 70 subjects in the 3 groups receiving SP and being screened for gametocytes on days 7, 10 and 14 respectively, and **c)** 130 subjects in the CQ group. All CQ- and CQ + SP-treated children will be screened for gametocytes on day 7 only. This will provide sufficient power at the 90% confidence level to detect a 10-fold difference in infectiousness at day 7 between gametocyte-positive children treated with SP alone, and those treated with SP + CQ.

**For studies involving human subjects:**

1. **How and where will the study subjects (cases, controls, etc) be selected? Has it been confirmed that they are not already involved in other studies?**

Subjects will be enrolled at Farafenni AFPRC Hospital/MCH clinic from among children presenting with uncomplicated falciparum malaria. Recruits will be randomised among the treatment groups.

1. **What inclusion/exclusion criteria will be applied?**

##### Children aged 1-10 years and the informed consent from a parent or guardian.

##### Inclusion Criteria

Fever or history of fever and a parasitaemia > 500/ul of *P.falciparum*

Informed consent from a parent or guardian.

Aged 1-10 years.

##### Exclusion Criteria

Signs or symptoms of severe malaria including severe anaemia (PCV <15% or Hb<5g/dL), or hyperparasitaemia (>500 parasites per hpf).

Vomiting or unable to take drugs orally.

A history of anti-malarial treatment in the past two weeks.

Evidence of any other chronic or acute illness.

1. **How will informed consent be obtained?**

Informed consent will be obtained from a parent or guardian at the time of enrolment into the study. The aims and methods, including details of blood samples needed, will be explained in a language they understand. The recruiting field worker/nurse will sign that consent was given. The right of all individuals not to participate or to withdraw their consent will be respected and the children will then receive standard treatment. Parents may withdraw their consent at any point in the trial and the child’s treatment will not be affected.

1. **What samples, if any, will be taken and what investigations will be conducted?**

- Finger-prick blood samples on days 0, 7, 14 and 28 to provide thick films for parasite detection and filter paper blood spots for molecular typing.
- Microtainer samples on day 0 for PCV, genotyping and RT-PCR
- 2-3ml blood by venepuncture on day 7 OR 10 OR 14 from consenting gametocyte-positive patients for membrane feeding of mosquitoes.
- Alcohol-fixed oocysts from mosquito guts for genotyping

1. **Will treatment be given? YES**

**If yes:**

**Nature of treatment(s)**

For drugs: dosage and duration of treatment

- A single dose of sulphadoxine /pyrimethamine (Fansidar®): for children ≤ 10kg, 0.5 tablet of SP (12.5mg and 250mg respectively), with an additional quarter tablet for each additional 5kg.
- Chloroquine at a total dose of 25mg/kg over 3 days with a single dose of sulphadoxine /pyrimethamine (Fansidar®) as above.
- Chloroquine at a total dose of 25mg/kg over 3 days.
- Each child will also be given a stat. dose of paracetamol (10 mg/kg) at enrolment.

Children will be observed for one hour for vomiting. Any child who vomits the study medication within one hour will receive a second dose. Children who vomit a second time will be dropped from the trial and treated with sub-cutaneous chloroquine.

Person(s) responsible for administering treatment

- Farafenni malaria epidemiologist and other MRC clinicians

1. **For questionnaires/interviews, who will be conducting these?**

- Field assistants and nurses

1. **Who will be primarily responsible for the statistical design and analysis?**

- The principle investigator in collaboration with LSHTM and MRC statisticians

1. **Who will be primarily responsible for data management?**

- The Farafenni data manager in collaboration with the principle investigator

**C Ethical issues**

**Please highlight any potential ethical issues and how you propose to deal with these. What outcomes and benefits will derive from the study? How will the results of the study contribute to the health of the people of the Gambia?**

This section, together with B3 is particularly important to the Ethical Committee and should be comprehensive. Please continue on a separate sheet if necessary.

This is a continuation of studies designed to establish the drug or drugs most appropriate for use in interrupting transmission of malaria to mosquitoes and the benefits gained by use of associating artemisinins with other anti-malarials. The primary benefit to the participants is that, as well as receiving free treatment, after treatment at the clinic they will be monitored by field assistants visiting their homes. In addition, the patients have access to study clinicians at the daily recruiting clinic run by the research team in the old Farafenni Health Centre, or at MRC Farafenni Field Station should the need arise.

The benefit of selecting a drug(s) that prevent transmission comes with its use for mass treatment of a population prior to the transmission season. The expectation is that this would significantly reduce the source of malaria infection in the subsequent malaria season, with a consequent reduction in malaria morbidity and mortality.

Collection of blood samples will be done only after informed consent has been obtained.

Any child in the study in need of additional medical attention will be provided with the appropriate treatment or referred.
