# Supplementary material for: Chloroquine/Sulphadoxine-Pyrimethamine for Gambian Children with Malaria: Transmission to Mosquitoes of Multidrug-Resistant Plasmodium falciparum
Source: PLoS Clin Trials. 2006 Jul 21;1(3):e15. doi: 10.1371/journal.pctr.0010015 (PMC1513405; doi:10.1371/journal.pctr.0010015)
Supplement: Table S1 — (343 KB DOC) [file pctr.0010015.st001.doc]

**Supplementary Table 1** Genotyping data from all feeds resulting in infected mosquito midguts

|  |  |  |  | **Day 0** | | | | | | | **Feed day** | | | | | | | **Midguts** | | | | | | | | |
| --- | --- | --- | --- | --- | --- | --- | --- | --- | --- | --- | --- | --- | --- | --- | --- | --- | --- | --- | --- | --- | --- | --- | --- | --- | --- | --- |
|  |  |  |  | **CQ resistance** | | | **SP resistance** | | | | **CQ resistance** | | | **SP resistance** | | | | **CQ resistance** | | | **SP resistance** | | | |  |  |
| **mf no.** | **ssn** | **feed day** | **drug** | **crt 76** | **mdr1 86** | **mdr1 184** | **dhfr 51** | **dhfr 59** | **dhfr 108** | **dhps 436/7** | **crt 76** | **mdr1 86** | **mdr1 184** | **dhfr 51** | **dhfr 59** | **dhfr 108** | **dhps 436/7** | **crt 76** | **mdr1 86** | **mdr1 184** | **dhfr 51** | **dhfr 59** | **dhfr 108** | **dhps 436/7** | **no. of oocysts** | **tube no.** |
| 21 | 160 | 7 | CQ | T | Y | F | N | C | S | SG | T | Y | F | N | C | S | SG | T | Y | F | N | C | S | SG | 9 | CS 2 |
| 40 | 323 | 7 | CQ | T | N | neg | I | R | N | AA | T | N | Y | NI | CR | N | AA/SA/SG | T | N | Y | I | CR | N | AA/SA | 2 | CS 1 |
|  |  |  |  |  |  |  |  |  |  |  |  |  |  |  |  |  |  | T | neg | neg |  |  |  |  | 10 | CS 11 |
|  |  |  |  |  |  |  |  |  |  |  |  |  |  |  |  |  |  | neg | neg | neg |  |  |  | SA | 21 | CS 2 |
|  |  |  |  |  |  |  |  |  |  |  |  |  |  |  |  |  |  | T | N | Y | NI | CR | N | AA/SA | 25 | CS 15 |
|  |  |  |  |  |  |  |  |  |  |  |  |  |  |  |  |  |  | T | N | Y | I | CR | N | AA/SA | 2 | CS 16 |
| 48 | 359 | 7 | CQ | KT | Y | F | I | R | N | SA/SG | T | NY | F | I | R | N | SA | T | Y | Y | N | C | N | SA | 4 | CS 8 |
| 50 | 367 | 7 | CQ | T | Y | F | I | R | N | AA | T | Y | F | I | R | N | SG | T | Y | F | I | R | N | SG | 1 | AP 3 |
|  |  |  |  |  |  |  |  |  |  |  |  |  |  |  |  |  |  | T | Y | F | I | R | N | SG | 3 | AP 4 |
| 62 | 451 | 7 | CQ | T | N | Y | N | C | S | SG | KT | NY | YF | N | C | SN | SG | T | N | Y | N | C | S | SG | 1 | CS 2 |
| 69 | 495 | 7 | CQ | T | Y | F | I | R | N | SA | T | Y | F | I | R | N | SA | neg | neg | neg |  |  |  |  | 6 | CS 1 |
|  |  |  |  |  |  |  |  |  |  |  |  |  |  |  |  |  |  | T | Y | F | I | R | N | SA | 36 | CS 2 |
|  |  |  |  |  |  |  |  |  |  |  |  |  |  |  |  |  |  | T | Y | F | I | R | N | SA | 100 | AP 1 |
|  |  |  |  |  |  |  |  |  |  |  |  |  |  |  |  |  |  | T | Y | F | I | R | N | SA | 100 | AP 2 |
| 16 | 102 | 7 | CQ/SP | T | Y | Y | I | R | N | SG | T | Y | Y | I | R | N | SG | neg | neg | neg |  |  |  |  | 1 | CS 1 |
|  |  |  |  |  |  |  |  |  |  |  |  |  |  |  |  |  |  | KT | N | YF | NI | CR | SN | AA/SA/SG | 1 | CS 10 |
| 27 | 218 | 7 | CQ/SP | T | Y | F | NI | CR | SN | SA/SG |  |  |  | I | R | N | AA/SG | T | Y | F | I | R | N |  | 3 | CS 9 |
|  |  |  |  |  |  |  |  |  |  |  |  |  |  |  |  |  |  | neg | neg | neg |  |  |  |  | 1 | CS 7 |
|  |  |  |  |  |  |  |  |  |  |  |  |  |  |  |  |  |  | neg | neg | neg |  |  |  |  | 1 | CS 15 |
|  |  |  |  |  |  |  |  |  |  |  |  |  |  |  |  |  |  | neg | neg | neg |  |  |  |  | 3 | CS 16 |
|  |  |  |  |  |  |  |  |  |  |  |  |  |  |  |  |  |  | neg | neg | neg |  |  |  |  | 1 | CS 19 |
|  |  |  |  |  |  |  |  |  |  |  |  |  |  |  |  |  |  | T | Y | F | I | CR | SN | SG | 3 | CS 18 |
|  |  |  |  |  |  |  |  |  |  |  |  |  |  |  |  |  |  | T | Y | F | I | R | N | SA/SG | 6 | CS 6 |
|  |  |  |  |  |  |  |  |  |  |  |  |  |  |  |  |  |  | neg | neg | neg |  |  |  |  | 2 | CS 17 |
|  |  |  |  |  |  |  |  |  |  |  |  |  |  |  |  |  |  | T | Y | F | I | R | N | SG | 9 | CS 10 |
| 49 | 360 | 7 | CQ/SP | neg | neg | neg | neg | neg | neg |  | K | Y | F | I | R | N | SA/SG | K | Y | F | I | R | N | SA | 5 | AP 8 |
|  |  |  |  |  |  |  |  |  |  |  |  |  |  |  |  |  |  | neg | neg | neg |  |  |  |  | 5 | AP 15 |
|  |  |  |  |  |  |  |  |  |  |  |  |  |  |  |  |  |  | K | Y | F | I | R | N | SA | 7 | AP 2 |
|  |  |  |  |  |  |  |  |  |  |  |  |  |  |  |  |  |  | K | Y | F | I | R | N | SA | 2 | AP 18 |
| 68 | 482 | 7 | CQ/SP | T | Y | F | NI | CR | SN | SG | T | Y | F | NI | CR | SN | SG | T | Y | F | I | R | N | SG | 39 | CS 6 |
|  |  |  |  |  |  |  |  |  |  |  |  |  |  |  |  |  |  | neg | Y | F |  |  |  |  | 2 | CS 10 |
|  |  |  |  |  |  |  |  |  |  |  |  |  |  |  |  |  |  | T | Y | F | I | R | N | SG | 11 | AP 4 |
|  |  |  |  |  |  |  |  |  |  |  |  |  |  |  |  |  |  | T | Y | F | I | R | N | SG | 29 | CS 7 |
|  |  |  |  |  |  |  |  |  |  |  |  |  |  |  |  |  |  | T | Y | F | I | R | N | SG | 14 | CS 9 |
|  |  |  |  |  |  |  |  |  |  |  |  |  |  |  |  |  |  | T | Y | F | I | R | N | SG | 34 | AP 2 |
|  |  |  |  |  |  |  |  |  |  |  |  |  |  |  |  |  |  | neg | neg | neg |  |  |  |  | 50 | AP 6 |
|  |  |  |  |  |  |  |  |  |  |  |  |  |  |  |  |  |  | T | Y | F | I | R | N | SG | 75 | CS 3 |
|  |  |  |  |  |  |  |  |  |  |  |  |  |  |  |  |  |  | T | Y | F | I | R | N | SG | 60 | AP 3 |
|  |  |  |  |  |  |  |  |  |  |  |  |  |  |  |  |  |  | T | Y | F | I | R | N | SG | 31 | CS 8 |
|  |  |  |  |  |  |  |  |  |  |  |  |  |  |  |  |  |  | T | Y | F | I | R | N | SG | 100 | CS 4 |
|  |  |  |  |  |  |  |  |  |  |  |  |  |  |  |  |  |  | T | Y | F | I | R | N | SG | 5 | CS 5 |
|  |  |  |  |  |  |  |  |  |  |  |  |  |  |  |  |  |  | T | Y | F | I | R | N | SG | 100 | AP 5 |
| 6 | 37 | 7 | SP | T | N | F | I | R | N | AA | T | Y | F | N | C | S | SG | T | N | F | I | R | N | AA | 4 | CS 1 |
| 13 | 66 | 10 | SP | KT | NY | F | NI | CR | SN | AA/SG | KT | NY | F | NI | CR | SN | AA/SG | neg | neg | neg |  |  |  |  | 3 | CS 4 |
|  |  |  |  |  |  |  |  |  |  |  |  |  |  |  |  |  |  | K | N | Y | N | R | N |  | 3 | CS 6 |
|  |  |  |  |  |  |  |  |  |  |  |  |  |  |  |  |  |  | neg | neg | neg |  |  |  |  | 1 | CS 10 |
| 56 | 365 | 14 | SP | T | neg | neg | I | R | N | SG | T | N | F | I | R | N | SG | T | N | F | I | R | N | SG | 9 | AP 11 |
|  |  |  |  |  |  |  |  |  |  |  |  |  |  |  |  |  |  | T | N | F | I | R | N | SG | 8 | AP 9 |
| 57 | 374 | 14 | SP | T | N | Y | I | R | N | SA | T | N | Y | I | R | N | SA | T | N | Y | I | R | N | SA | 17 | CS 9 |
|  |  |  |  |  |  |  |  |  |  |  |  |  |  |  |  |  |  | T | N | Y | I | R | N | SA | 8 | CS 3 |
|  |  |  |  |  |  |  |  |  |  |  |  |  |  |  |  |  |  | T | N | Y | I | R | N | SA | 36 | AP 9 |
|  |  |  |  |  |  |  |  |  |  |  |  |  |  |  |  |  |  | neg | N | Y | I | R | N | neg | 9 | CS 7 |
| 58 | 423 | 10 | SP | T | Y | F | I | R | N | SG | T | Y | F | I | R | N | SG | T | Y | F | I | R | N | SG | 1 | AP 3 |
|  |  |  |  |  |  |  |  |  |  |  |  |  |  |  |  |  |  | T | Y | F | I | R | N | AG | 2 | AP 4 |
|  |  |  |  |  |  |  |  |  |  |  |  |  |  |  |  |  |  | T | Y | F | I | R | N | AG | 4 | AP 1 |
|  |  |  |  |  |  |  |  |  |  |  |  |  |  |  |  |  |  | midgut missing |  |  |  |  |  |  | 1 | AP 2 |
| 59 | 416 | 10 | SP | T | Y | Y | I | R | N |  | T | Y | Y | I | R | N | AA | neg | neg | neg |  |  |  | AG | 3 | CS 5 |
|  |  |  |  |  |  |  |  |  |  |  |  |  |  |  |  |  |  | T | Y | F | I | R | N | AG | 1 | AP 3 |
|  |  |  |  |  |  |  |  |  |  |  |  |  |  |  |  |  |  | T | Y | F | I | R | N | AG | 2 | CS 1 |
|  |  |  |  |  |  |  |  |  |  |  |  |  |  |  |  |  |  | T | Y | Y | I | R | N | AG | 50 | CS 3 |
|  |  |  |  |  |  |  |  |  |  |  |  |  |  |  |  |  |  | neg | neg | neg |  |  |  |  | 1 | CS 4 |
|  |  |  |  |  |  |  |  |  |  |  |  |  |  |  |  |  |  | T | Y | F | I | R | N | AG | 3 | AP 2 |
|  |  |  |  |  |  |  |  |  |  |  |  |  |  |  |  |  |  | T | Y | F | I | R | N | AG | 3 | AP 9 |
|  |  |  |  |  |  |  |  |  |  |  |  |  |  |  |  |  |  | T | Y | F | I | R | N | AG | 8 | AP 8 |
|  |  |  |  |  |  |  |  |  |  |  |  |  |  |  |  |  |  | T | Y | F | I | R | N | AG | 2 | CS 6 |
| 67 | 447 | 14 | SP | T | Y | F | I | R | N |  | T | Y | F | I | R | N | SG | T | Y | F | I | R | N | AG | 2 | CS 4 |
